# Mutual mate choice and its benefits for both sexes

**DOI:** 10.1038/s41598-020-76615-z

**Published:** 2020-11-10

**Authors:** Alicia Reyes-Ramírez, Iván Antonio Sandoval-García, Maya Rocha-Ortega, Alex Córdoba-Aguilar

**Affiliations:** Departamento de Ecología Evolutiva, Instituto de Ecología, Universidad Nacional Autónoma de México, Apdo. P. 70-275, Circuito Exterior, Ciudad Universitaria, 04510 Coyoacán, Distrito Federal Mexico

**Keywords:** Ecology, Evolution, Physiology, Zoology

## Abstract

In mating interactions, it is common in nature for both sexes to choose simultaneously. However, this mutual mate choice and its consequences for progeny has received relatively little study; an approach where both male and female condition is manipulated is thus desirable. We compared both sexes’ preferences in *Tenebrio molitor* beetles when individual condition varied (healthy vs infected with a fungus), and observed the direct benefits of those preferences. We predicted that: (a) females and males in good condition would prefer high quality mates; (b) preferences would be weaker when the choosing individual is in poor condition (and thus less selective given, for example, time and energetic constrains); and, (c) high quality mates would lay a larger number of total eggs and/or viable eggs than low quality mates. We found that both males and females in good condition were not more likely to choose mates that were also in good condition. However, poor-condition animals were more likely to prefer similar quality animals, while high-condition animals did not necessarily prefer mates of similar condition. Choosing sick males or females had a negative impact on egg number and viability. Our results suggest a non-adaptive mate choice in this species. Possibly, a deteriorated condition may drive individuals to invest more in attracting mates, because their chances of surviving the infection are very low. However, we do not discount the possibility that the fungus is manipulating individuals to increase its transmission during mating.

## Introduction

The choosy sex is the one that invests more in reproduction and broad evidence has demonstrated that frequently this is the case for females rather than males^1–4^. In this way, females choose males that can offer them a series of benefits, both direct and indirect^5,6^. Conversely, males produce a large number of less costly gametes and are limited almost exclusively by their mating rate, and therefore should compete for mates^2,3,7^. While these roles are widely accepted, male choosiness is also possible^8^. We have some evidence that there may be limitations on male reproductive capacity (e.g. sperm production) and therefore male choice is expected because the costs males incur in reproduction, though substantially less than those of females, are not trivial^9,10^. Cases of male choice were first documented in species where males invest substantially in parental care^11–13^. However, it is now known that male choice can also occur in the absence of male parental care^12,14,15^. Male mate choice evolves as long as males benefit from being choosy, for example by incurring a significant cost during courtship and/or mating, or by detecting variation in the quality of females^9,12,16^. Given this situation, perhaps the best way to understand and accept mate choice is to see it as mutual^8^.

Mutual mate choice and its effects on offspring has been studied relatively little when the condition (broadly defined here as the general health and vigor of an organism^17^) of the two sexes differs. In these situations, it is expected that both sexes will choose mates of higher quality to obtain the corresponding benefits^18,19^. One example is that of the crayfish *Procambarus clarkii*, where both males and females prefer larger mates^20^. While females obtain the benefit of mating with larger males because they provide refuges necessary during reproduction, males benefit from mating with larger females due to the positive correlation between female size and fecundity^20^. In fact, male choice of more fecund females is a recurring pattern in an enormous variety of taxonomic groups where female size is the indicator^21–23^. It is worth pointing out that the benefits for the two sexes need not be the same. For most species, the benefit for males of choosing a high-quality mate is a larger number of offspring^23,24^. On the other hand, in the case of females, the benefit of mating with a high-quality male is having more attractive and/or viable offspring^4,25,26^. In this way, a pair consisting of a high-quality male and female should give rise to a larger number of more attractive and/or viable young (see for example^19^).

One way to evaluate mate quality is through ornaments that act as honest signals of condition^27–30^. An aspect of condition related to the expression of ornaments is the intensity of transmissible parasites, which indicate not only resistance to infections^31,32^, but also the risk of acquiring a parasite during mating^33–36^. One type of ornament is sex pheromones. These compounds are chemical signals that are transmitted by diffusing through the air or water, and are effective even though they are emitted at low concentrations^37,38^. Pheromones fulfill all of the criteria to be considered indicators of good condition, and therefore act as ornaments for the following reasons: (1) their quantity and/or quality vary among individuals of the same species^39,40^, (2) they honestly reflect individual quality^37,41^, (3) they are costly both to produce and to maintain (i.e. their production is reduced when the animal is nutrient-stressed^42,43^), and (4) they show relatively high levels of heritability^39^.

In insects, the production of pheromones to attract the opposite sex is a fairly generalized phenomenon^44–46^. One example is the case of the mealworm, *Tenebrio molitor*, where it has been demonstrated that females evaluate males based on males’ pheromones^37,47,48^. In this species, 3‐dodecenyl acetate is the main component of male pheromones^49^, but other cuticular hydrocarbons play some role^50^. Intriguingly, variation in male condition using a diversity of immune challenges have given rise to inconsistent findings on mate choice. On the one hand, it has been documented that females were more attracted to healthy males^51,52^, however, other works have reported that they also preferred sick males^50,53–56^. Furthermore, the only study that investigated the consequences for preferring the pheromones of sick males on the offspring found that females that mated with sick males had less viable eggs than females that mated with healthy males^56^. Interestingly, female *T. molitor* also produce pheromones, but in their case composed of 4 methyl-1-nonanol^49^. Notwithstanding, we know of no study that has evaluated the role of these compounds as a reflection of female condition, their role in male choice, or their benefits for males.

In this study, we investigated mutual mate choice and its benefits when both sexes vary in their condition, using the evaluation of pheromones and *T. molitor* as a study subject. We used the entomopathogenic fungus *Metarhizium robertsii* (formerly known as *Metarhizium anisopliae*) to induce changes in the condition of both sexes. This fungus is a natural pathogen of this insect^57^ as well as of other species (as many as 600 species)^58–60^. The genus *Metarhizium* is cosmopolitan and is found in soils, where it commonly comes in contact with *T. molitor*^61,62^. In a pre-copulatory context, pheromone production is the only critical perceived difference between good and poor-quality males. We evaluated the benefits of choice in both sexes in terms of egg number and hatching success. Our predictions were: (1) individuals in good condition (non-infected males and females) would prefer individual mates in good condition, due to increased investment in pheromone production; (2) poor condition individuals (infected with the fungus) would be less selective (given that they have less time or energy to choose compared to high-quality individuals^19,63,64^) than good condition individuals (uninfected males and females); and, 3) matings of uninfected males and females would yield higher numbers of eggs and higher viability than pairings between a male or female infected with the fungus.

## Materials and methods

### Insect breeding

A *T. molitor* colony was formed with specimens from five different breeding centers from Mexico City and the State of Mexico. The colony had approximately 5000 specimens. Larvae were fed ad libitum with wheat bran (Maxilu brand) as food and apple every week as a source of water. The colony was kept at room temperature conditions (25 ± 2 °C) with a 12-h photoperiod (12 light hours/12 dark hours). The pupae were sexed using morphological structures located in the eighth abdominal segment^65^. The sexes were kept separately in plastic containers (22.1 cm length × 15.4 cm width × 5.7 cm height; 100 individuals per container) to prevent mating before the choice experiment. Adults were fed the same diet as when they were larvae.

### Preparation of the fungus

The fungus *M. robertsii* (ARSEF 2134) was obtained through the Entomopathogenic Fungi Collection of the Agricultural Research Service of the United States Department of Agriculture. Spores were transported in a 10% glycerol solution at − 80 °C and placed for storage on Sabouraud Dextrose Agar (SDA) to be incubated later for 15 days at 28 °C without light exposure. Conidiophores were then carefully collected from the plate and suspended in 0.03% Tween 80. Tween 80 (polysorbate 80) is one of the most favorable surfactants for the propagation of microorganisms and conidia of different fungi of medical importance and for pest control^66–68^. The suspension was mixed by vortexing for 5 min and filtered through a cotton mesh to separate the conidia from the mycelium. Conidia number was counted, and their percentage viability was determined using a Neubauer chamber.

### Determination of the LC_50_

We had five different *M. robertsii* conidia concentrations which were suspended in 10 ml of Tween (1 × 10^4^, 1 × 10^5^, 1 × 10^6^, 1 × 10^7^, and 1 × 10^8^ conidia/ml). Fifteen males and fifteen females per concentration were inoculated by submerging insects in the suspension for 5 s. As far as we are aware, there is no information of how insects get infected by this fungus in the field but contact between the insect and fungus is via either soil or conspecific contact^58^. Animals used in this process were aged between 12 and 15 days and had a weight range of 0.09–0.12 g. Animals were then placed in groups on Whitman No. 1 filter paper inside a 9-cm diameter Petri dish. A control group was submerged in Tween without fungus and dried in the same way as the infected groups. Insects were placed separately in 12-well plates with wheat bran and incubated at 25 °C and 90% humidity for 10 days. Mortality (assessed as animals that did not move even after being manipulated) was recorded every 24 h. Dead insects were removed and placed on wet filter paper in order to stimulate sporulation and confirm the infection. LC_50_ was estimated as 3.9 × 10^5^ conidia/ml, confidence interval of 95% from 8.11 × 104 years to 1.43 × 106 conidia/ml. All steps were carried out on sterilized surfaces. Please note that this LC_50_ is similar to that documented in previous studies and whose survival indicates that fungus treated individuals die before the other groups^56^.

### Treatments and their application

Three treatments were applied to both sexes 12–15 days after individuals reached the adult stage. At this age, beetles have already reached sexual maturity^69^. The treatments were as follows: (1) a fungus-infected group—individuals were infected with the fungus by immersion for 5 s in a dilution of Tween 80 at 0.03% with a *M. robertsii* LC_50_; (2) Tween control group—animals were immersed for 5 s in Tween 80 at 0.03%; and, (3) Non-manipulated control group—animals were treated in the same ways as the other two groups except that they were not infected nor immersed in Tween. After applying the treatments, individuals were placed separately in 12-well plates supplied with wheat bran and kept in an incubator at 25 °C 3 days until the day of their choice test. It should be noted that individuals infected with the fungus do not present any type of sporulation during the choice tests. There was no apparent decrease in mobility when compared to control treatments groups.

### Female and male choice trials

#### Generalities

**Figure 1:**
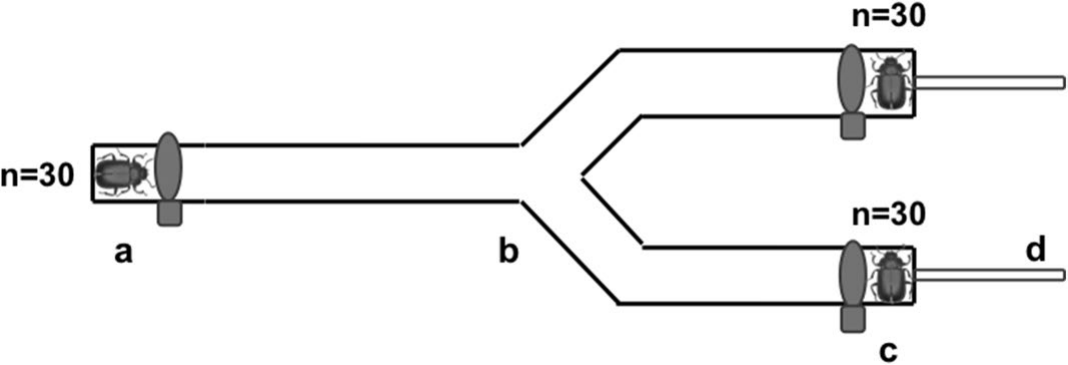
Example of one assay of mate choice in a Y olfactometer, where sample sizes are shown. (**a**) Release port where the choosing individual is placed; (**b**) section where the individual makes its choice; (**c**) arms where the individuals to choose are placed; and, (**d**) connections with the air stream.

Mate choice trials were carried out in a "Y" shaped olfactometer fitted with an air pump connected to each arm. The olfactometer was made of glass and had three gates that allowed air circulation from the arms to the release port, preventing beetles from having physical contact with each other. After each test, the olfactometer was cleaned with ethanol to reduce the accumulation of pheromones and residual chemicals. The tests were conducted inside a dark room with a 100-W red spotlight that cannot be detected by and thus affect the beetles but allowed us to record their choice^70^. In total we carried out 18 choice assays, nine for female choice and nine for male choice (see Fig. 1). In each female choice assay, n = 30 females and n = 60 males (30 males from one treatment vs. 30 males from a different treatment) were used. As for each male choice assay, n = 30 males and n = 60 females from two different treatments were used (30 females from one treatment vs. 30 females from a different treatment). In each trial, an individual chose between an individual of the same health status of the choosing one vs. an individual of a different health status to the choosing one. All individuals were used once. The observer was not aware of the health status of either the choosing individual or their two choices.

#### Experimental trials

For female choice trials, a female was placed at the release port, while a male from one treatment was placed in arm 1 and another male from a different treatment in arm 2 (see Fig. 1). The location of each male was randomly assigned. Females were acclimatized for 2 min, then the gate was opened for the female to choose one of the males among the experimental combinations indicated above. Choice trials covered all possible combinations with females of each condition (fungus infected, Tween control and non-manipulated), choosing among males from the three treatments (fungus infected, Tween control and non-manipulated).

For male choice tests, the roles of the two sexes were inverted in the olfactometer, with the females located in the arms, and the males in the release port. The same combinations of male and female condition were used as indicated above. The time it took males to choose and the side they preferred was also recorded.

### Effects on offspring

The pairs formed from each mate choice trial were placed in plastic containers (6.1 cm in diameter and 4.6 cm high) with 9 g of wheat flour (Tres Estrellas brand) for 1 week. Flour facilitates egg extraction and provides nutrients during this period. After a week of egg laying, flour was sieved using a Montinos no. 60 sieve to collect the eggs. Eggs were then kept in open tubes and were placed back in flour to record hatching rate for 3 weeks.

### Statistical analyses

A generalized linear mixed-effect model with a binomial error distribution was used to evaluate the effect of the following independent fixed variables on the preferences during mutual choice and their interactions: sex of the choosing individual, sex of the chosen individual, health status of the choosing individual (either male of or female) and health status of the chosen individual. Trials were entered as a random factor, that is, the three individuals which belonged to the same status health condition. Through a stepwise regression strategy via backward selection (or backward elimination), we chose the best model based on the lowest Akaike Information Criterion (AIC) values. We started entering all independent variables to then remove the ones with the lowest contribution. Notice that a significant difference was only found in the interaction between the health status of the choosing individual and the health status of the chosen individual. To assess whether the number of eggs and proportion of eggs hatched were related to the health status of females and males, two independent generalized linear models were used for each mate choice. Both the total number of eggs and the proportion of hatched eggs were used as dependent variables, while the health status of the different partners was set as an independent variable. A Poisson and binomial distribution were used for the number of eggs and hatching rate, respectively. Tukey tests were used to compare the differences between treatments. Furthermore, to assess the influence of males and females on egg number, the effect sizes were obtained. In mixed models effect size is measured as semi partial R^2^ values with confidence limits and is helpful for summarizing model goodness-of-fit. All analyses were done in R^71^ with the “lme4” and “r2glmm” packages^72,73^.

## Results

### Mutual mate choice

Regardless of sex, males and females did not differ in their preferences (χ^2^ = 0.0667, *P* > 0.05). Health status of the choosing individual had an effect on his/her choice (χ^2^ = 18.5092, *P* < 0.001; Table 1). A larger number of couples where both sexes were infected with the fungus were obtained than couples where both individuals were healthy (z = − 2.198, *P* < 0.05). However, there were more couples formed by Tween individuals compared to couples formed by individuals infected with the fungus (z = 2.282, *P* < 0.05).

**Table 1:** Summary of generalized linear modelling (GLM), generalized linear mixed modelling (GLMM), and their effects sizes.

	GLM/GLMM			Effect size		
	df	χ^2^	*P*	Semi-partial R^2^	Upper CL	Lower CL
**Mate choice**				0.295	0.664	0.19
Health status of the choosing individual	2	0.0003	> 0.05			
Health status of the chosen individual	2	0.667	> 0.05			
Health status of the choosing individual × health status of the chosen individual	4	18.509	**< 0.001**			
**Eggs by female choice**				0.276	0.402	0.197
Parents’ health status	8	739.33	**< 0.001**			
**Hatching success by female choice**				0.133	0.264	0.082
Parents’ health status	8	319.62	**< 0.001**			
**Eggs by male choice**				0.222	0.352	0.15
Parents’ health status	8	876.52	**< 0.001**			
**Hatching success by male choice**				0.222	0.352	0.15
Parents’ health status	8	76.428	**< 0.001**			

Significant differences appear in bold.

### Effects on offspring of female choice

**Figure 2:**
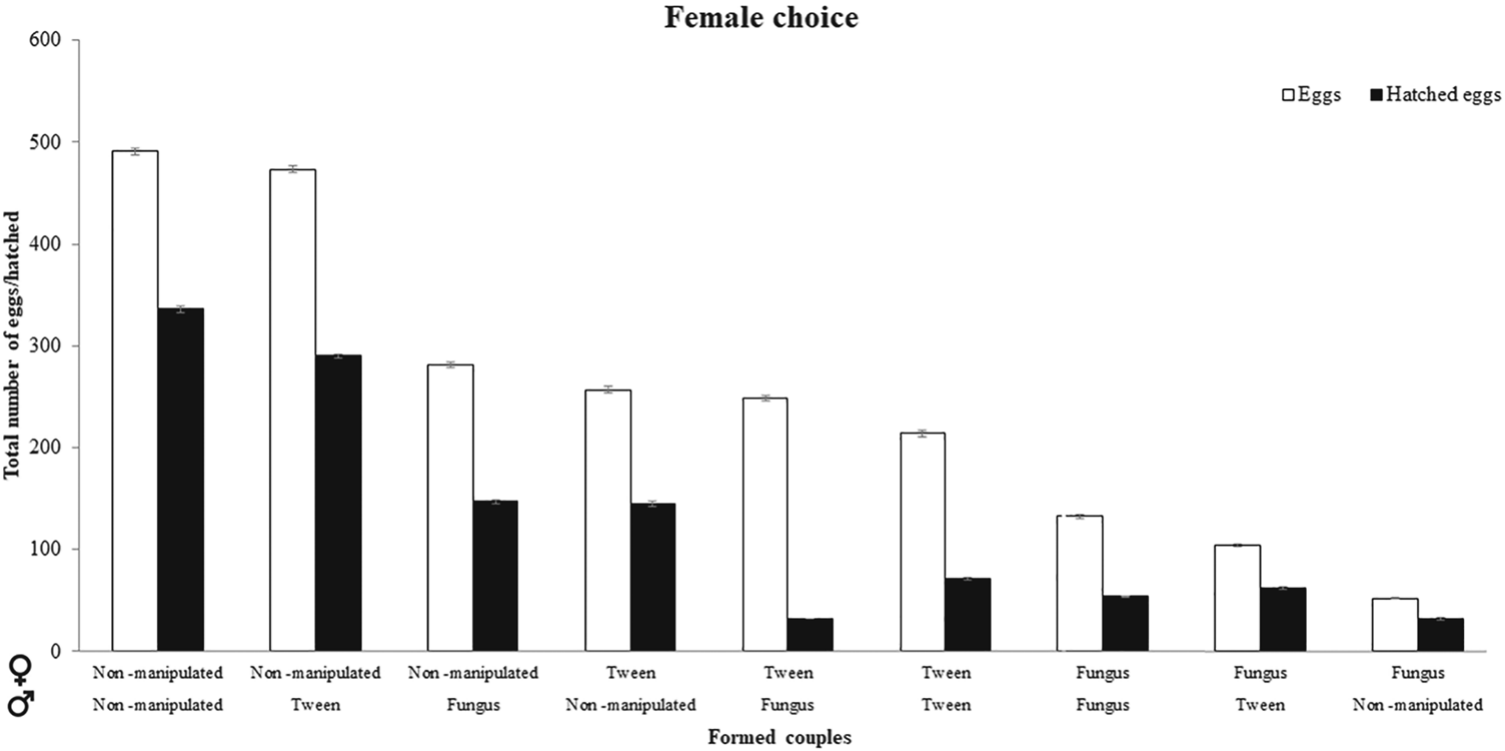
Number of eggs laid (white bars) and hatching success (dark bars) according to all female-male pairing combinations and in regard to experimental condition (non-manipulated, Tween control, and fungus-treated) after female choice. Horizontal labels indicate name of treatment for female (top) and male (below). For example, the first pairing combination indicates a non-manipulated female and a non-manipulated male.

The health status of both parents affected the number of eggs laid by the pairs (χ^2^ = 739.33, *P* < 0.001; Fig. 2). The pairs that had the most eggs were composed of non-manipulated males and non-manipulated females. The pairs that had the fewest eggs were composed of an infected female and non-manipulated male (for all combinations see supplementary material Table S1). The effects size also showed that females had a greater impact than males on the number of eggs (Table 1).

The health status of females and males also affected the hatching success of the eggs (χ^2^ = 319.62, *P* < 0.001; Fig. 2). Hatching success was highest among the eggs of non-manipulated males and females. On the other hand, eggs belonging to Tween control females and infected males had the lowest hatching success (for all combinations see supplementary material Table S2).

### Effects on offspring of male choice

**Figure 3:**
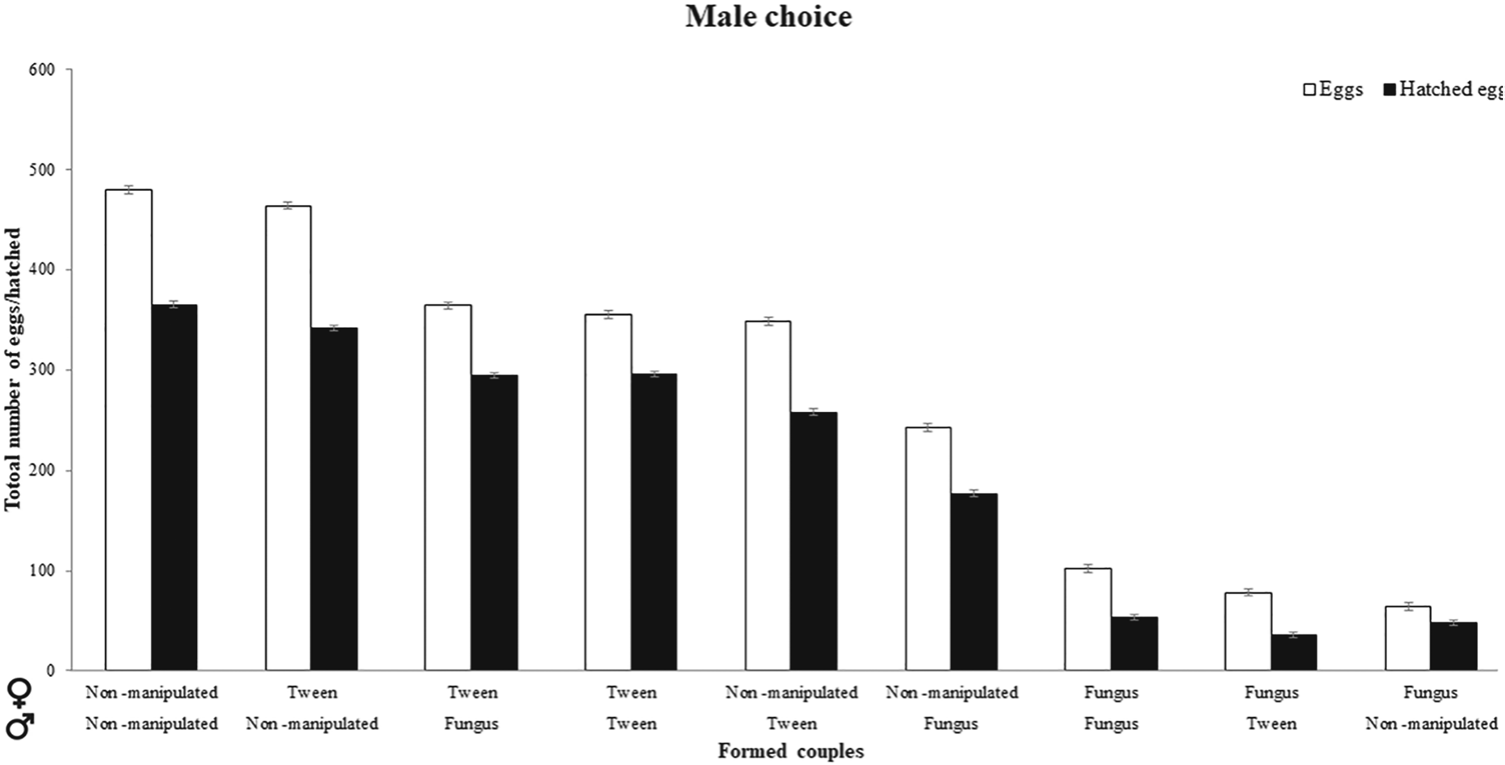
Number of eggs laid (white bars) and hatching success (dark bars) according to all female-male pairing combinations and in regard to experimental condition (non-manipulated, Tween control, and fungus-treated) after male choice. Horizontal labels indicate name of treatment for female (top) and male (below). For example, the first pairing combination indicates a non-manipulated female and a non-manipulated male.

The health status of both sexes affected the number of eggs laid by the different pairs (χ^2^ = 876.52, *P* < 0.001; Fig. 3). Again, the pairs comprising a non-manipulated male and non-manipulated female had the largest number of eggs, and the pairs with a non-manipulated male and infected female had the lowest number of eggs (for all combinations see supplementary material Table S3).

The health status of the males and females also affected hatching success (χ^2^ = 76.428, *P* < 0.001; Fig. 3). When males were the choosing sex, the pairs with a Tween control male and Tween control female had the highest hatching success (for all combinations see supplementary material Table S4). The pairs with the lowest success were Tween control males and fungus-infected females (see supplementary material Table S4).

## Discussion

In general, the results of our mate choice trials and their benefits are not entirely compatible with our predictions (i.e. that high-quality individuals will prefer high-quality mates while poor-quality individuals would be less selective). We first predicted that high-quality females and males will choose mates of a similar quality which was not corroborated. Conversely, we found that manipulated (fungus infected and Tween) individuals were more likely to choose mates of equal condition. One explanation is that the pairs dedicate all of their resources to the production of pheromones to attract mates more intensely. This explanation, widely known as terminal investment^74,75^ has been described in a wide variety of taxonomic groups^76^ including our study species^50,53–55^. According to these studies, in *T. molitor*, fungus-infected males die faster than control males^56^, which corroborates the idea of terminal investment. While the two sexes of *T. molitor* produce different pheromones^44^, theoretically males and females of the same species could perform terminal investment when under attack from a pathogen^76^. This logic may well explain why fungus-infected and Tween individuals attracted more mates of a similar quality. However, it does not explain why manipulated individuals tended to choose mates in an assortative fashion (i.e. fungus infected animals with fungus infected animals). It is as if the nature of a challenge (whether fungus infected or Tween) drives the mate choice criteria for similar mates. It may also be that the fungus manipulated the choosing individuals to choose other fungus infected animals as partners, but this would not explain why Tween individuals chose Tween animals as these are not infected.

One notable finding from our mate choice experiment is that mutual choice would produce different combinations of mate phenotypes. There are not many studies with which to compare our results because, unlike other studies, we varied the condition of both sexes. In this sense, other studies of male mate choice have found that males prefer females with more intense ornaments^14,77,78^, but not when condition has been modified in both sexes. In fact, our experiments are closer to natural contexts in which both pairs choose and are confronted with an enormous diversity of options with respect to quality^79–81^. In the case of *T. molitor*, where both sexes live in colonies where there is intense competition for resources^82,83^, encounters between the sexes, and therefore the opportunity to choose mates, are extremely common^84^. However, if we apply our findings to understand mate choice in natural conditions, then mate preferences seem non-adaptive and may drive a population invaded by a pathogen to extinction. Whether this is driven by a terminal investment situation or fungus manipulation, a deeper analysis is required at the population level.

The results of the direct benefits of mate choice for both sexes are also in partial agreement with our predictions (i.e. that matings between uninfected individuals would produce more eggs and higher viability than matings when one partner is infected). We found that when both members of the pair are in good condition, there were more offspring, and in the case of female choice, increased survival of the offspring. These results indicate that when both sexes are in good condition, they benefit from mating with equally healthy mates, as corroborated by a plethora of studies^85–88^. Male choice was less clear with respect to benefits. These results also indicate that even when males and females may be undergoing terminal investment and thus investing more intensely to attract a mate, there is a penalty to the mate that allows itself to be “seduced”. These results had already been reported in *T. molitor* and suggest a lack of correlation between the expression of an ornament, in this case pheromones, and the quality of the progeny^56^. Moreover, our results also suggest that quality of progeny is mainly influenced by females rather than males. This is similar to results in vertebrates, in which progeny mass is reduced when mothers are subjected to an immune challenge^89^. As such, the fact that the combination of control males and infected females had eggs with lower hatching rates could have been due to decreased investment in lipids by females. Finally, it is notable that the pairs consisting of Tween and infected individuals had the fewest eggs and lowest survival. This suggests that the Tween treatment is not ideal, but unfortunately, we have not found a way to reduce the dose in a way that allows the use of the fungus treatment.

Considering the results of mate choice as well as the consequences for offspring, it is evident that the two sexes do not necessarily coincide on how they invest their resources. The possibility of terminal investment, which is an interesting constant in this species^50,53–55^, involves fitness costs. Given this, it is difficult to explain why mechanisms have not evolved to discriminate males and females whose investment in pheromones does not correspond with direct benefits. As indicated before, one explanation is that the fungus manipulates the infected individuals in order to facilitate its dispersal among individuals, as predicted by the idea of parasite-host coevolution^90^. This should be tested in future studies. One other explanation is that there may be additional cues that both partners can use that allow for further mate discrimination during mate choice. Related to this, *T. molitor* males intensively court during mating^91,92^. Courting aspects include leg and antennal movements mainly carried out by the male making contact with the female’s body^91–93^. This courtship behavior has not been assessed in terms of mate choice and benefits but it may also act as another cue that females may use for biasing their resources allocated to offspring. The fact that we left the couples that were formed using only pheromone-based preferences, did not allow us to test this hypothesis. However, further tests using females that have mated with males of varying quality can allow for the investigation of whether male courtship plays a role in mate choice and fitness benefits.

## Supplementary information

Supplementary Tables.
