# Supplementary material for: Mutual mate choice and its benefits for both sexes
Source: Sci Rep. 2020 Nov 10;10:19492. doi: 10.1038/s41598-020-76615-z (PMC7656247; doi:10.1038/s41598-020-76615-z)
Supplement: Supplementary file 1 — Supplementary Tables. [file 41598_2020_76615_MOESM1_ESM.docx]

Table S1 Comparisons between the possible couples formed in the female choice according to the health status of both sexes and their effect on the number of eggs laid according to the Tukey test

| Treatments | Estimate Std. | SE | z value | p |
| --- | --- | --- | --- | --- |
| fn-ff | -0.905 | 0.020 | -43.885 | **< 0.001** |
| ft-ff | -0.268 | 0.016 | -15.952 | **< 0.001** |
| nf-ff | 0.725 | 0.013 | 53.813 | **< 0.001** |
| nn-ff | 1.283 | 0.012 | 102.623 | **< 0.001** |
| nt-ff | 1.246 | 0.012 | 99.229 | **< 0.001** |
| tf-ff | 0.600 | 0.013 | 43.613 | **< 0.001** |
| tn-ff | 0.636 | 0.013 | 46.490 | **< 0.001** |
| tt-ff | 0.512 | 0.013 | 36.595 | **< 0.001** |
| ft-fn | 0.637 | 0.021 | 29.597 | **< 0.001** |
| nf-fn | 1.631 | 0.019 | 85.684 | **< 0.001** |
| nn-fn | 2.189 | 0.018 | 119.253 | **< 0.001** |
| nt-fn | 2.151 | 0.018 | 116.994 | **< 0.001** |
| tf-fn | 1.506 | 0.019 | 78.273 | **< 0.001** |
| tn-fn | 1.541 | 0.019 | 80.382 | **< 0.001** |
| tt-fn | 1.417 | 0.019 | 73.064 | **< 0.001** |
| nf-ft | 0.993 | 0.014 | 67.079 | **< 0.001** |
| nn-ft | 1.552 | 0.013 | 111.373 | **< 0.001** |
| nt-ft | 1.514 | 0.013 | 108.334 | **< 0.001** |
| tf-ft | 0.869 | 0.015 | 57.622 | **< 0.001** |
| tn-ft | 0.904 | 0.015 | 60.298 | **< 0.001** |
| tt-ft | 0.780 | 0.015 | 51.062 | **< 0.001** |
| nn-nf | 0.558 | 0.009 | 57.792 | **< 0.001** |
| nt-nf | 0.520 | 0.009 | 53.554 | **< 0.001** |
| tf-nf | -0.124 | 0.011 | -11.107 | **< 0.001** |
| tn-nf | -0.089 | 0.011 | -8.012 | **< 0.001** |
| tt-nf | -0.213 | 0.011 | -18.523 | **< 0.001** |
| nt-nn | -0.037 | 0.008 | -4.490 | **< 0.001** |
| tf-nn | -0.683 | 0.010 | -67.913 | **< 0.001** |
| tn-nn | -0.647 | 0.009 | -65.130 | **< 0.001** |
| tt-nn | -0.771 | 0.010 | -74.456 | **< 0.001** |
| tf-nt | -0.645 | 0.010 | -63.793 | **< 0.001** |
| tn-nt | -0.610 | 0.010 | -60.975 | **< 0.001** |
| tt-nt | -0.734 | 0.010 | -70.429 | **< 0.001** |
| tn-pf | 0.035 | 0.011 | 3.102 | **< 0.05** |
| tt-tf | -0.088 | 0.011 | -7.461 | **< 0.001** |
| tt-tn | -0.124 | 0.011 | -10.556 | **< 0.001** |

In the first column all combinations of couples are shown according to the health status of males and females. On the left is the treatment of the male and on the right that of the female, for example in the first combination Male: Fungus infected / Female: Non-manipulated vs Male: Fungus infected / Female: Fungus infected. Significant effects (p <0.05) are in bold.

Table S2 Comparisons between the possible couples formed in the female choice according to the health status of both sexes and their effect on the hatching of eggs laid according to the Tukey test

| Treatments | Estimate Std. | SE | z value | p |
| --- | --- | --- | --- | --- |
| fn-ff | 0.620 | 0.041 | 14.870 | **< 0.001** |
| ft-ff | 0.900 | 0.034 | 26.114 | **< 0.001** |
| nf-ff | 0.603 | 0.027 | 21.879 | **< 0.001** |
| nn-ff | 1.284 | 0.026 | 49.259 | **< 0.001** |
| nt-ff | 0.971 | 0.025 | 37.482 | **< 0.001** |
| tf-ff | -1.398 | 0.033 | -41.778 | **< 0.001** |
| tn-ff | 0.769 | 0.028 | 27.421 | **< 0.001** |
| tt-ff | -0.558 | 0.030 | -18.530 | **< 0.001** |
| ft-fn | 0.280 | 0.043 | 6.461 | **< 0.001** |
| nf-fn | -0.016 | 0.038 | -0.436 | 0.999 |
| nn-fn | 0.664 | 0.037 | 17.934 | **< 0.001** |
| nt-fn | 0.351 | 0.036 | 9.508 | **< 0.001** |
| tf-fn | -2.018 | 0.042 | -47.402 | **< 0.001** |
| tn-fn | 0.149 | 0.038 | 3.875 | **< 0.01** |
| tt-fn | -1.178 | 0.040 | -29.451 | **< 0.001** |
| nf-ft | -0.296 | 0.030 | -9.877 | **< 0.001** |
| nn-ft | 0.384 | 0.028 | 13.395 | **< 0.001** |
| nt-ft | 0.070 | 0.028 | 2.486 | 0.222 |
| tf-ft | -2.299 | 0.035 | -64.674 | **< 0.001** |
| tn-ft | -0.131 | 0.030 | -4.305 | **< 0.001** |
| tt-ft | -1.458 | 0.032 | -44.989 | **< 0.001** |
| nn-nf | 0.681 | 0.019 | 34.274 | **< 0.001** |
| nt-nf | 0.367 | 0.019 | 18.713 | **< 0.001** |
| tf-nf | -2.002 | 0.028 | -69.256 | **< 0.001** |
| tn-nf | 0.165 | 0.022 | 7.397 | **< 0.001** |
| tt-nf | -1.162 | 0.024 | -46.529 | **< 0.001** |
| nt-nn | -0.313 | 0.017 | -17.919 | **< 0.001** |
| tf-nn | -2.683 | 0.027 | -97.645 | **< 0.001** |
| tn-nn | -0.515 | 0.020 | -25.125 | **< 0.001** |
| tt-nn | -1.843 | 0.023 | -79.089 | **< 0.001** |
| tf-nt | -2.369 | 0.027 | -86.738 | **< 0.001** |
| tn-nt | -0.202 | 0.020 | -9.956 | **< 0.001** |
| tt-nt | -1.529 | 0.023 | -66.171 | **< 0.001** |
| tn-pf | 2.167 | 0.029 | 73.846 | **< 0.001** |
| tt-tf | 0.840 | 0.031 | 26.781 | **< 0.001** |
| tt-tn | -1.327 | 0.025 | -52.087 | **< 0.001** |

In the first column all combinations of couples are shown according to the health status of males and females. On the left is the treatment of the male and on the right that of the female, for example in the first combination Male: Fungus infected / Female: Non manipulated vs Male: Fungus infected / Female: Fungus infected. Significant effects (p <0.05) are in bold.

Table S3 Comparisons between the possible couples formed in the male choice according to the health status of both sexes and their effect on the number of eggs laid according to the Tukey test

| Treatments | Estimate Std. | SE | z value | p |
| --- | --- | --- | --- | --- |
| fn-ff | 0.868 | 0.015 | 56.995 | **< 0.001** |
| ft-ff | 1.272 | 0.014 | 87.959 | **< 0.001** |
| nf-ff | -0.466 | 0.020 | -22.640 | **< 0.001** |
| nn-ff | 1.546 | 0.014 | 109.868 | **< 0.001** |
| nt-ff | 1.514 | 0.014 | 107.303 | **< 0.001** |
| tf-ff | -0.268 | 0.019 | -13.815 | **< 0.001** |
| tn-ff | 1.227 | 0.014 | 84.428 | **< 0.001** |
| tt-ff | 1.247 | 0.014 | 85.990 | **< 0.001** |
| ft-fn | 0.404 | 0.010 | 37.785 | **< 0.001** |
| nf-fn | -1.334 | 0.018 | -73.555 | **< 0.001** |
| nn-fn | 0.678 | 0.010 | 66.745 | **< 0.001** |
| nt-fn | 0.646 | 0.010 | 63.272 | **< 0.001** |
| tf-fn | -1.136 | 0.016 | -67.637 | **< 0.001** |
| tn-fn | 0.359 | 0.010 | 33.277 | **< 0.001** |
| tt-fn | 0.379 | 0.010 | 35.265 | **< 0.001** |
| nf-ft | -1.738 | 0.017 | -99.337 | **< 0.001** |
| nn-ft | 0.274 | 0.008 | 30.584 | **< 0.001** |
| nt-ft | 0.242 | 0.009 | 26.853 | **< 0.001** |
| tf-ft | -1.540 | 0.016 | -95.633 | **< 0.001** |
| tn-ft | -0.044 | 0.009 | -4.644 | **< 0.001** |
| tt-ft | -0.025 | 0.009 | -2.600 | 0.170 |
| nn-nf | 2.012 | 0.017 | 117.149 | **< 0.001** |
| nt-nf | 1.981 | 0.017 | 115.078 | **< 0.001** |
| tf-nf | 0.197 | 0.021 | 9.086 | **< 0.001** |
| tn-nf | 1.693 | 0.017 | 96.437 | **< 0.001** |
| tt-nf | 1.713 | 0.017 | 97.721 | **< 0.001** |
| nt-nn | -0.031 | 0.008 | -3.783 | **< 0.01** |
| tf-nn | -1.814 | 0.015 | -115.143 | **< 0.001** |
| tn-nn | -0.319 | 0.009 | -35.136 | **< 0.001** |
| tt-nn | -0.299 | 0.009 | -33.135 | **< 0.001** |
| tf-nt | -1.783 | 0.015 | -112.869 | **< 0.001** |
| tn-nt | -0.287 | 0.009 | -31.424 | **< 0.001** |
| tt-nt | -0.267 | 0.009 | -29.415 | **< 0.001** |
| tn-pf | 1.495 | 0.016 | 92.468 | **< 0.001** |
| tt-tf | 1.515 | 0.016 | 93.869 | **< 0.001** |
| tt-tn | 0.019 | 0.009 | 2.045 | 0.485 |

In the first column all combinations of couples are shown according to the health status of males and females. On the left is the treatment of the male and on the right that of the female, for example in the first combination Male: Fungus infected / Female: Non manipulated vs Male: Fungus infected / Female: Fungus infected. Significant effects (p <0.05) are in bold.

Table S4 Comparisons between the possible couples formed in the male choice according to the health status of both sexes and their effect on the hatching of eggs laid according to the Tukey test

| Treatments | Estimate Std. | SE | z value | p |
| --- | --- | --- | --- | --- |
| fn-ff | 0.908 | 0.031 | 28.696 | **< 0.001** |
| ft-ff | 1.356 | 0.030 | 44.029 | **< 0.001** |
| nf-ff | 1.020 | 0.045 | 22.567 | **< 0.001** |
| nn-ff | 1.085 | 0.029 | 37.301 | **< 0.001** |
| nt-ff | 0.952 | 0.289 | 32.859 | **< 0.001** |
| tf-ff | -0.232 | 0.389 | -5.978 | **< 0.001** |
| tn-ff | 0.974 | 0.030 | 32.411 | **< 0.001** |
| tt-ff | 1.514 | 0.031 | 48.150 | **< 0.001** |
| ft-fn | 0.448 | 0.025 | 17.712 | **< 0.001** |
| nf-fn | 0.112 | 0.041 | 2.691 | 0.139 |
| nn-fn | 0.177 | 0.023 | 7.636 | **< 0.001** |
| nt-fn | 0.044 | 0.023 | 1.920 | 0.579 |
| tf-fn | -1.140 | 0.034 | -32.839 | **< 0.001** |
| tn-fn | 0.066 | 0.024 | 2.729 | 0.126 |
| tt-fn | 0.060 | 0.026 | 23.228 | **< 0.001** |
| nf-ft | -0.336 | 0.041 | -8.200 | **< 0.001** |
| nn-ft | -0.271 | 0.022 | -12.302 | **< 0.001** |
| nt-ft | 0.404 | 0.021 | -18.451 | **< 0.001** |
| tf-ft | -1.589 | 0.033 | -46.771 | **< 0.001** |
| tn-ft | -0.381 | 0.023 | -16.367 | **< 0.001** |
| tt-ft | 0.157 | 0.025 | 6.282 | **< 0.001** |
| nn-nf | 0.065 | 0.039 | 1.637 | 0.769 |
| nt-nf | -0.067 | 0.039 | -1.709 | 0.724 |
| tf-nf | -1.252 | 0.047 | -26.418 | **< 0.001** |
| tn-nf | -0.045 | 0.040 | -1.123 | 0.968 |
| tt-nf | 0.494 | 0.041 | 11.901 | **< 0.001** |
| nt-nn | -0.132 | 0.019 | -6.843 | **< 0.001** |
| tf-nn | -1.317 | 0.032 | -40.638 | **< 0.001** |
| tn-nn | -0.110 | 0.021 | -5.260 | **< 0.001** |
| tt-nn | 0.428 | 0.022 | 18.700 | **< 0.001** |
| tf-nt | -1.184 | 0.032 | -36.653 | **< 0.001** |
| tn-nt | 0.022 | 0.020 | 1.072 | 0.976 |
| tt-nt | 0.561 | 0.022 | 24.647 | **< 0.001** |
| tn-pf | 1.207 | 0.033 | 36.244 | **< 0.001** |
| tt-tf | 1.746 | 0.034 | 50.550 | **< 0.001** |
| tt-tn | 0.539 | 0.024 | 22.323 | **< 0.001** |

In the first column all combinations of couples are shown according to the health status of males and females. On the left is the treatment of the male and on the right that of the female, for example in the first combination Male: Fungus infected / Female: Non manipulated vs Male: Fungus infected / Female: Fungus infected. Significant effects (p <0.05) are in bold.
